# Healthy eating index 2015 and major dietary patterns in relation to incident hypertension; a prospective cohort study

**DOI:** 10.1186/s12889-022-13166-0

**Published:** 2022-04-13

**Authors:** Yahya Pasdar, Behrooz Hamzeh, Shima Moradi, Ehsan Mohammadi, Sahar Cheshmeh, Mitra Darbandi, Roya Safari Faramani, Farid Najafi

**Affiliations:** 1 Department of Nutritional Sciences, Research Center for Environmental Determinants of Health (RCEDH), Health Institute, Kermanshah University of Medical Sciences, Kermanshah, Iran; 2 Research Center for Environmental Determinants of Health (RCEDH), Health Institute, Kermanshah University of Medical Sciences, Kermanshah, Iran; 3 Department of Occupational Health Engineering, Faculty of Health, Qazvin University of Medical Sciences, Qazvin, Iran; 4 Student research committee, School of Nutritional Sciences and Food Technology, Kermanshah University of Medical Sciences, Kermanshah, Iran; 5 School of Public Health, Kermanshah University of Medical Sciences, Kermanshah, Iran; 6 Department of Epidemiology, School of Health, Social Development and Health Promotion Research Center, Research Institute for Health, Kermanshah University of Medical Sciences, Kermanshah, Iran; 7 School of Public Health, Communing Developmental and Health Promotion Research Center, Kermanshah University of Medical Sciences, Kermanshah, Iran

**Keywords:** Diet, Hypertension, Incidence, Dietary sodium

## Abstract

**Background:**

Since hypertension (HTN) is responsible for more than half of all deaths from cardiovascular disease, it is vital to understand the nutritional factors that reduce its risk. Little information, however, is known about it in the Kurdish population. This study was aimed to evaluate the healthy eating index (HEI) 2015 and major dietary patterns concerning incident HTN.

**Methods:**

This case-cohort study was designed using Ravansar non-communicable diseases (RaNCD) cohort study data (294 participants with incident HTN and 1295 participants as representative random sub-cohort). HEI 2015 and major dietary patterns were extracted using data from their dietary intake, and three major dietary patterns were identified, including plant-based, high protein, and unhealthy dietary patterns. To analyses the association between HEI 2015 and major dietary patterns with incident HTN Cox proportional hazards regression models were applied.

**Results:**

There was a significant positive correlation between HEI 2015 and plant-based diet (*r* = 0.492). The participants in the highest quartile of HEI-2015 had a 39% and 30% lower risk of incident HTN, compared to participants in the first quartile in both crude and adjusted model (HR: 0.61; 95% CI: 0.46–0.82) and (HR: 0.70; 95% CI: 0.51–0.97), respectively. Furthermore, participants with the highest tertile of the plant-based dietary pattern were at lower risk of incident HTN in both crude and adjusted models (HR: 0.69; 95% CI: 0.54–0.9) and (HR: 0.70; 95% CI: 0.53–0.94), respectively. However, the other two identified dietary patterns showed no significant association with incident HTN.

**Conclusions:**

We found evidence indicating higher adherence to HEI 2015 and plant- based diet had protective effects on incident HTN. The HEI 2015 emphasizes limited sodium intake and adequate intake of vegetables and fruits.

## Background

Hypertension (HTN) is a global health problem, the principal cause of cardiovascular diseases (CVDs) and premature death, affecting roughly one billion adults worldwide [1, 2]. By 2030, it is estimated that 41% of American adults will have been diagnosed with HTN [3]. In contrast to the trends reported in the USA and northern Europe, evidence indicates that CVDs mortality is rising in Iran, and 24% of females and 22% of males suffer from HTN [4, 2]. HTN is a multifactorial disease caused by a complex combination of dietary, lifestyle, and genetic risk factors [5]. The high prevalence of HTN can be attributed to sedentary behaviour, obesity, smoking, family history, and an unwholesome diet [2, 5, 6]. Interestingly, not only is it a curable and well-modifiable disease the controlled HTN but also improves the life quality and the prognosis and prevents relating clinical complications [2, 7]. The main approaches for preventing and managing HTN are lifestyle changes, involving physical activity, weight loss, and dietary modifications [2, 8, 9].

Dietary modifications play a crucial role in preventing and managing HTN[1]. Numerous studies have been conducted over the past few decades that highlight the importance of dietary modification in developing and controlling HTN [8–11]. Several indices have recently been developed to examine the healthiness of dietary patterns. Due to the interaction of nutrients in foods, it is often impossible to differentiate the effects of specific dietary ingredients. Accordingly, the usage of these indices and dietary patterns has evolved to assess the effects of overall dietary intake [10, 12, 13]. There is also the “food group effect”. Food intake is multidimensional exposure, namely food and component effect, contaminants, additives. Assessment of dietary patterns increases the ability to assess more substantial effects due to the cumulative effects of many diet factors (inter-correlations), and allow assessing of the interaction among synergistic components. Since 1995 the healthy eating index (HEI) has been a dietary index developed to evaluate the diet's quality in accordance with Dietary Guidelines for Americans (DGA). The HEI is updated every five years by the evidence-based recommendations of the United States Department of Agriculture (USDA) and Health and Human Services (HHS) [14, 15]. The latest version of this index is the HEI 2015 index noting two essential features in nutrition guidelines, adequacy moderation for dietary intakes. The first part consists of 9 food items, including total fruits, whole fruits, total vegetables, greens and beans, whole grains, dairy, total protein foods, seafood and plant proteins, and fatty acids and the second part that means limitation intake is related to following four food items, refined grains, sodium, added sugars, and saturated fats [14, 16].

Despite the tremendous increase in the prevalence of HTN in Iran [17], there are no standard quantitative dietary guidelines to evaluate dietary patterns and their relationships with HTN [5]. Additionally, little information about the relation between HTN and HEI 2015 and major dietary patterns in the Kurdish population is known. The Ravansar Non-Communicable Disease cohort study [18] provided this opportunity to estimate dietary patterns and HTN associations from an epidemiological standpoint. Therefore, the current study investigated the HEI 2015 and major dietary patterns in relation to incident HTN.

## Methods

### Study design and participants

This present case-cohort study was applied to data from the RaNCD cohort study in which the Kurdish population-based study was started as a prospective study for evaluating non-communicable diseases in October 2014 in Ravansar, Kermanshah Province, western Iran. The RaNCD cohort study is a part of the Prospective Epidemiological Research Studies in Iran (PERSIAN) mega cohort study with 10,047 participants (4764 men and 5283 women) aged 35–65 years in which recruitment phase commenced from October 2014 to January 2017 and followed until January 2021. Ethical approval for the RaNCD cohort study has been granted by the Ethics Committee of Kermanshah University of Medical Sciences, Kermanshah, Iran (No: KUMS.REC.1394.318), and written informed consent was completed from all participants prior to contribution. The protocols of these studies have already been published [18, 19].

Initially, 1295 participants without HTN incident were randomly considered as representative sub-cohort (20% of the base population). Among the rest, participants with hypertension were excluded (*n* = 1452), CVDs (*n* = 512), thyroid (*n* = 139), and cancer (*n* = 18) diseases, as well as pregnant women (*n* = 134) due to possible dietary changes. Additionally, incident CVDs (*n* = 134) were excluded from this study. Furthermore, the participants whose calories intake was not in the normal range, (800–4200 kcal/day for men and 600- 3500 kcal/day for women) (*n* = 796), were eliminated from the study. Finally, 294 participants with incident HTN remained and were selected as the case group.

### Data sources/ measurements

We obtained data required for this analysis from the RaNCD cohort study, including demographics, socioeconomic factors and current lifestyles, such as physical activity (metabolic equivalent of task (MET) hour per day), educational level, smoking status (never, current, or former smoker), anthropometric indices, dietary intake, and medical history. The details of measurement methods are described in the study of the RaNCD cohort profile [18].

### Anthropometric measurements

The height and weight of participants were measured with the least clothing and without shoes by the automatic stadiometer BSM 370 (Biospace Co., Seoul, Korea) and InBody 770 device (Inbody Co, Seoul, Korea), respectively in the study site in Ravansar in a standing position. Waist circumference (WC) measuring was applied using non-stretched and flexible tape in a standing position at the level of the iliac crest. Body mass index (BMI) was computed by dividing weight in kg into height square in meters.

### Healthy eating index 2015

The study included adults with complete and reliable dietary data from national validated semi-quantitative 118 items food frequency questionnaire (FFQ) published in the previous studies [18, 20]. HEI 2015 was calculated based on RaNCD FFQ data using the method described by Krebs-Smith et al. [16]. HEI 2015 include 13 items, including whole fruits, total fruits, total protein foods, total vegetables, seafood and plant proteins, greens and beans, whole grains, dairy products, fatty acids, refined grains, sodium, added sugars and saturated fats. These food items were divided into two categories of adequacy and moderation food groups. Adequacy diet components contain whole fruits, total fruits, whole protein foods, total vegetables, seafood and plant proteins, greens and beans, whole grains, dairy products, and fatty acids, which is encouraged to increase intake, and a higher score reflects adequate intake. On the other hand, in HEI 2015, it is recommended to limit the intake of moderate diet components (the last four are refined grains, sodium, added sugars and saturated fats), and a higher score indicates that their intake has been limited. For scoring the first six, the amount consumed is given a point from zero to 5 and the rest of the range of points is from zero to ten. The score from each item, ultimately, is added together and the final HEI score is between zero and 100. (Table 1).

**Table 1:** Healthy eating index—2015^1^

Component	Standard for maximum score ^1^	Standard for minimum score of zero	Maximum points
Adequacy:			
** Total Fruits**^2^	≥ 0.8 cup equivalent per 1,000 kcal	No Fruit	5
** Whole Fruits**^3^	≥ 0.4 cup equivalent per 1,000 kcal	No Whole Fruit	5
** Total Vegetables**^4^	≥ 1.1 cup equivalent per 1,000 kcal	No Vegetables	5
** Greens and Beans**^4^	≥ 0.2 cup equivalent per 1,000 kcal	No Dark-Green Vegetables or Legumes	5
** Whole Grains**	≥ 1.5 cup equivalent per 1,000 kcal	No Whole Grains	10
** Dairy**^5^	≥ 1.3 cup equivalent per 1,000 kcal	No Dairy	10
** Total Protein Foods**^4^	≥ 2.5 cup equivalent per 1,000 kcal	No Protein Foods	5
** Seafood and Plant Proteins**^4,6^	≥ 0.8 cup equivalent per 1,000 kcal	No Seafood or Plant Proteins	5
** Fatty Acids**^7^	(PUFAs + MUFAs)/SFAs ≥ 2.5	(PUFAs + MUFAs)/SFAs ≤ 1.2	10
Moderation:			
** Refined Grains**	≤ 1.8 oz equivalent per 1,000 kcal	≥ 4.3 oz equivalent per 1,000 kcal	10
** Sodium**	≤ 1.1 g per 1,000 kcal	≥ 2.0 g per 1,000 kcal	10
** Added Sugars**	≤ 6.5% of energy	≥ 26% of energy	10
** Saturated Fats**	≤ 8% of energy	≥ 16% of energy	10
Total score			100

^1^ Intakes between the minimum and maximum standards are scored proportionately

^2^ Includes 100% fruit juice

^3^ Includes all forms except juice

^4^ Includes legumes (beans and peas)

^5^ Includes all milk products, such as fluid milk, yogurt, and cheese, and fortified soy beverages

^6^ Includes seafood, nuts, seeds, soy products (other than beverages), and legumes (beans and peas)

^7^ Ratio of poly- and mono-unsaturated fatty acids (PUFAs and MUFAs) to saturated fatty acids (SFAs)

### Dietary pattern

The major dietary patterns were identified by principal component analysis to energy-adjusted foods intake using data from the RaNCD FFQ. At the beginning, we categorized all food items considering nutrients similarity into 31 food groups. (Table 2) In the method of principal component analysis, the varimax rotation was applied to create a distinct and straightforward matrix and kept uncorrelated factor variables called the major pattern. The scree-plot was also drawn to determine the number of matrix components (the major dietary patterns)..The typical interpretation of the eigen values greater than 1 and the Scree diagram implied that three factor should remain. The extracted factors, dietary patterns, were identified based on the recent studies. The factor score of each dietary pattern was computed by calculating the factor load from every group dietary intake. Food groups with a factor loading exceeding 0.2 were used to correlate between food groups and the known dietary pattern. Participants individually received a score per pattern based on factor scores and then categorized into tertiles according to dietary model scores.

**Table 2:** Food groupings used in the dietary pattern analyses

Food groups	Dietary components
Leafy vegetables	Cauliflower, lettuce, cucumber, onion, green bean, mushroom, pepper, garlic, turnip, others
Fresh fruits	Melon, watermelon, honeydew melon, plums, prunes, apples, cherries, sour cherries, peaches, nectarine, pear, fig, date, grapes, kiwi, pomegranate, strawberry, banana, persimmon, berry, pineapple, oranges, others
Dried fruits	Dried apricots, Dried berries, raisins, and other type dried fruits
Dairy	Milk, yogurt, yogurt drink (doogh), cheese, chocolate milk, crud
Tomato	Tomato
Carotene-rich vegetables	Yellow squash, carrot
Condiments	Condiments
Pickles	Pickles
Legumes	All type beans, peas,lentils, mung bean, soy
Whole grain	Dark breads (Iranian), wheat, barley
Starchy vegetables	Corn, eggplant, green peas, green squash
Vegetable oil	Vegetable oil
Natural juices	All fruit juices
Butter	Butter, margarine, mayonnaise
Olive	Olive and olive oil
Organ meat	Heart, kidney, liver, tongue, brain, offal
Read meat	Beef, lamb, minced meat
Fish	All fish types
Processed meat	Hamburger, sausage, delicatessen meat, pizza
Soft drink	Soft drink
Nuts	Almond, peanut, walnut, pistachio, hazelnut, seeds
Egg	Egg
Poultry	Chicken
Snack	Corn puffs, potato chips, French fries
Sweets and desserts	Cookies, cakes, biscuit, muffins, pies, chocolates, ice, honey, jam, sugar cubes, sugar, candies, others
Tea and coffee	Tea and coffee
Hydrogenated fat	Hydrogenated fats, animal fats
Salt	Salt
Potato	Potato
Refined grain	White breads (lavash, baguettes), noodles, pasta, rice

### Blood pressure

The systolic and diastolic blood pressure (SBP and DBP) were measured after at least 4–5 min of rest by conventional sphygmomanometer and auscultation of the Korotkoff sounds in a sitting position two times with 10 min interval, and the mean of them was reported as the final blood pressure [18].

### Hypertension (HTN) incidence

The incident HTN was defined based on the codes I10 of the International classification of diseases Tenth Edition (ICD-10), SBP/DBP ≥ 140/90 mmHg and/or using anti-hypertensive medications in the time interval between baseline (the first phase of Ravansar cohort has been conducted from 2014) and hypertension diagnosis (from 2015 to 2021), which the overall duration of the follow-up was 850.26 person-year, with a median duration of follow up of 2.32 years (min: 0.5, max: 4.8).

### Statistical analysis

All statistical analysis was performed using Stata, version 14 (Stata Corp, College Station, TX) with *P*-value < 0.05 as a significant level. Differences in the baseline quantitative and qualitative variables across quartile of HEI 2015 were assessed using the One-way analysis of variance (ANOVA) and Chi -square tests, respectively. Correlation between major identified dietary patterns and HEI 2015 was determined by Person correlation and the difference of HEI 2015 across tertiles of major identified dietary patterns was evaluated by the One-way ANOVA.

Cox proportional hazards regression model were used to calculate hazard ratios (HRs) stratified by HEI 2015 and major identified dietary patterns, with hypertension as the event and the time interval between baseline (first phase of RaNCD cohort) and hypertension diagnosis as the time covariate. The fundamental assumption in the Cox model is that the hazards are proportional (proportional hazards), which means that the relative hazard remains constant over time with different predictor or covariate levels. The models were adjusted for confounding variables, including Modell I: unadjusted; Model II: adjusted for sex (categorical) and age (continuous); Model III: adjusted for Model II plus socioeconomic status (SES) (categorical), education levels (categorical), physical activity (continuous), diabetes (categorical); Model IV: adjusted for Model III plus BMI (continuous) and energy intake (continuous) and finally reported as HR with 95% confidence interval (CI). We also assessed the incidence of HTN per HEI-2015 components in crude and adjusted model for all mentioned adjusted variables in the previous analysis.

## Results

The mean age was 46.5 ± 7.89 years and 48.83% were male. The representative random sub-cohort of 1295 participants was selected for this case-cohort study in the current prospective study. After exclusions, 294 incidents of HTN occurred. The mean weight, BMI, and WC in cases were significantly higher than in the sub-cohort group. In addition, the mean PA of cases was remarkably lower than in the sub-cohort group (*P* < 0.001). There was no significant difference between two studied groups regarding gender (*P* < 0.001). Baseline characteristics of the study population in both studied groups are shown in Table 3.

**Table 3:** Baseline characteristics in cases and sub cohort groups

Determinants	Total (*n* = 1587)	Cases (*n* = 294)	Sub cohort (*n* = 1291)
**Age (year)**	46.5 ± 7.89	50.43 ± 7.34	45.59 ± 7.74
**Gender, male, %**	48.83	38.1	51.35
**SES, %**^1^			
Weak	33.18	36.52	32.38
Moderate	33.43	31.4	33.85
Good	33.37	32.08	33.77
**Weight (kg)**	72.25 ± 13.07	73.61 ± 13.3	71.94 ± 13
**BMI** ^2^**(kg/m**^**2**^**)**	27.23 ± 4.42	28.47 ± 4.35	26.95 ± 4.39
**WC**^3^ **(cm)**	96.67 ± 10.03	99.33 ± 9.39	96.06 ± 10.07
**PA** ^4^ **(MET/ day)**	40.99 ± 8.18	39.77 ± 7.2	41.27 ± 8.37
**Energy intake (Kcal/day)**	2518.97 ± 689.9	2416.42 ± 721.05	2541.25 ± 716.19
**Smoking, %**	18.54	19.59	18.31
**Diabetes, %**	6.5	5.34	11.64
**Components of HEI**			
**Total Fruits**	2.96 ± 1.32	2.93 ± 1.31	3.07 ± 1.36
**Whole Fruits**	4.08 ± 1.23	4.07 ± 1.24	4.12 ± 1.17
**Total Vegetables**	3.63 ± 1.11	3.62 ± 1.11	3.68 ± 1.12
**Greens and Beans**	3.43 ± 1.27	3.41 ± 1.27	3.49 ± 1.26
**Whole Grains**	1.42 ± 1.05	1.41 ± 1.04	1.44 ± 1.12
**Dairy**	4.99 ± 2.81	4.97 ± 2.84	4.99 ± 2.80
**Total Protein Foods**	3.15 ± 1.12	3.04 ± 1.13	3.18 ± 1.12
**Seafood and Plant Proteins**	4.18 ± 0.69	4.17 ± 0.68	4.19 ± 0.72
**Fatty Acids**	4.97 ± 3.07	4.96 ± 3.06	5.01 ± 3.10
**Refined Grains**	0.20 ± 0.99	0.18 ± 0.94	0.30 ± 1.18
**Sodium**	2.42 ± 2.73	2.31 ± 2.68	2.45 ± 2.74
**Added Sugars**	8.84 ± 1.89	8.72 ± 2.13	8.87 ± 1.83
**Saturated Fats**	7.17 ± 2.68	7.13 ± 2.71	7.17 ± 2.67
**Total HEI score**	51.42 ± 7.38	51.41 ± 7.38	51.48 ± 7.40

^1^*SES* Socioeconomic status,^2^ *BMI* Body mass index, ^3^ *WC* Waist circumference, ^4^ *PA* Physical activity

The food groups intake results determined three major dietary patterns: plant-based, high protein, and unhealthy. The plant- based diet was characterized by higher adherence to leafy vegetables, starchy vegetables, carotene-rich vegetables, tomato, potato, legumes, nuts, olive, vegetable oil, fresh and dried fruits, and fruit juice. Another major dietary pattern, a high protein diet, tended to a higher intake of red and white meat, legumes, egg, whole, and refined grains. In addition, unhealthy dietary pattern was specified with a higher factor loading of salt, sweet, dessert, butter, hydrogenated fat, soft drink, refined grain, tea, and coffee. (Table 4).

**Table 4:** Factor loading of food groups in all dietary patterns

Food groups	Plant- based dietary pattern	High protein dietary pattern	Unhealthy dietary pattern
**Leafy vegetables**	.684	-	-
**Fresh fruits**	.637	.221	
**Pickles**	.491	-	.205
**Starchy vegetables**	.445	-	-
**Condiments**	.441	-	.300
**Dried fruits**	.436	-	-
**Tomato**	.407	-	-
**Carotene-rich vegetables**	.396	.268	
**Nuts**	.393	.214	-
**Vegetable oil**	.380	-	-.341
**Dairy**	.357	-	-
**Natural juices**	.336	.224	-
**Organ meat**	-	.665	-
**Red meat**	-	.637	-
**Fish**	-	.545	-
**Processed meat**	-	.448	-
**Legumes**	.293	.423	-
**Poultry**	-	.373	-
**Soft drinks**	-	.365	.339
**Egg**	-	.342	-
**Refined grains**	-	.327	.254
**Whole grains**	.218	.262	-
**Sweets and desserts**	-	-	.658
**Hydrogenated fats**	-	-	.561
**Tea and coffee**	-	-	.544
**Salt**	-	-	.353
**Potato**	.246	-	.311
**Butters**	.234	.231	.300
**Olive**	.272	-	-.275
**Snack**	.213	-	.263
**Variance %**	13.72	6.55	5.4

Values < 0.2 have been removed for clarity

Table 5 shows the correlation between HEI 2015 and the major identified dietary patterns. According to it, there was a significant positive correlation between HEI 2015 with plant-based (*r* = 0.492) and high protein (*r* = 0.255) diets, while there was a notable inverse correlation between HEI 2015 and unhealthy diet (*r* = -0.473).

**Table 5:** The correlation between healthy eating index 2015 and major identified dietary patterns

Major dietary pattern	Categories	HEI	P*	r	P**
		Mean ± SD			
**Plant- based dietary pattern**	T1	46.65 ± 6.06	< 0.001	0.492	< 0.001
	T2	51.91 ± 6.14			
	T3	55.68 ± 6.91			
**High protein dietary pattern**	T1	48.8 ± 7.23	< 0.001	0.255	< 0.001
	T2	51.63 ± 6.68			
	T3	53.83 ± 7.34			
**Unhealthy dietary pattern**	T1	55.5 ± 6.86	< 0.001	-0.473	< 0.001
	T2	50.85 ± 6.63			
	T3	47.91 ± 6.56			

P* was obtained one way ANOVA

P** was obtained Pearson correlation

**Fig. 1:**
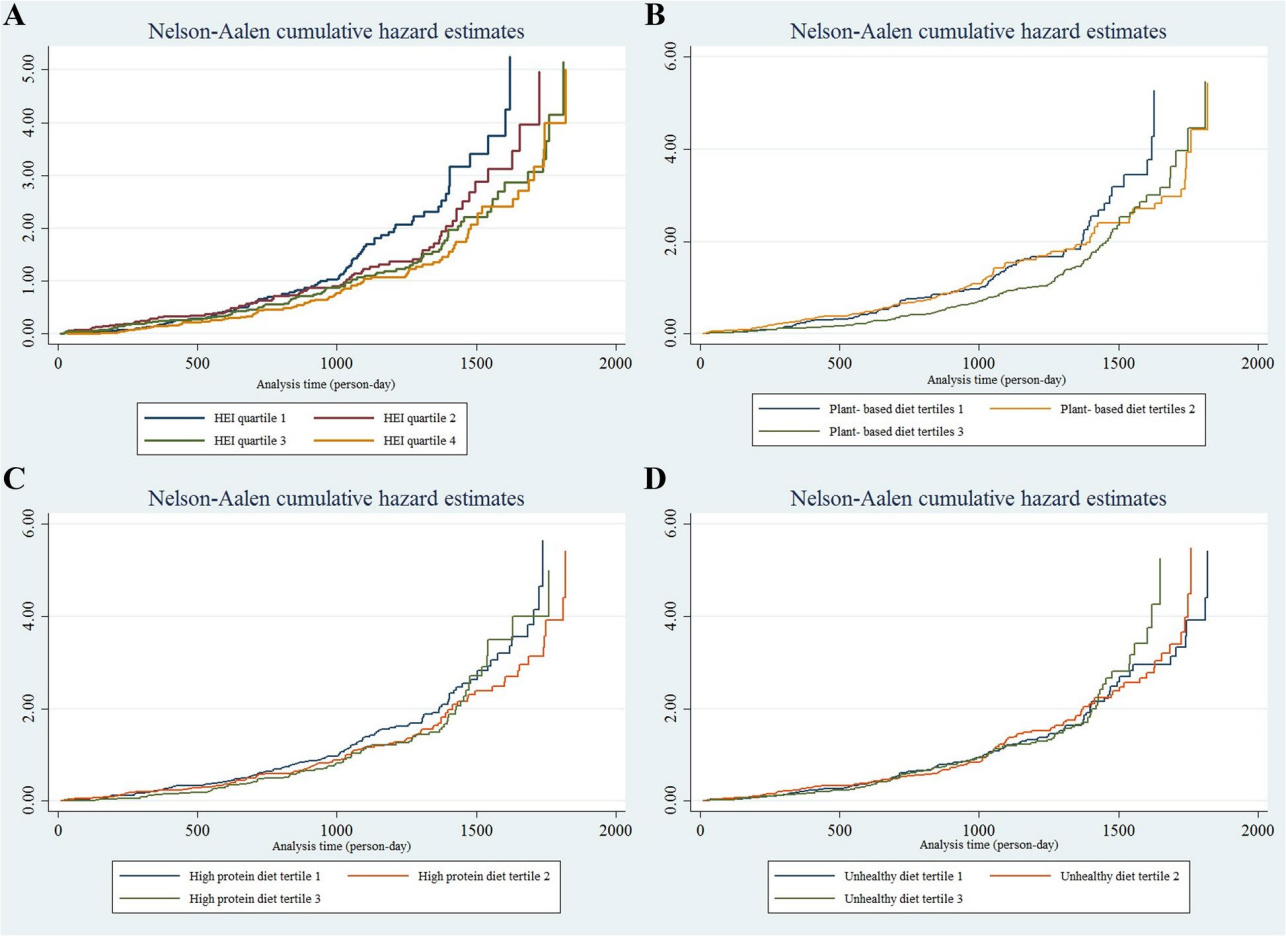
Estimates of the cumulative baseline hazard functions for the hypertension data by **A**: HEI; **B**: plant- based diet; **C**: high protein diet; **D**: unhealthy diet

This present study revealed that the individuals in the highest quartile of HEI-2015 had a 39% lower risk of incident HTN than the participants in the first quartile (Model I: HR: 0.61; 95% CI: 0.46–0.82). After adjustment for potential confounders, including age, gender, SES, education, physical activity, diabetes, BMI, and energy intake, this association was remained (Model IV: HR: 0.7; 95% CI: 0.51–0.97). (Table 6) (Fig. 1, A) Similarly, among the major identified dietary patterns, persons who were the highest tertile of the plant-based dietary pattern were at lower risk of incident HTN in both crude and adjusted models (Model I: HR: 0.69; 95% CI: 0.54–0.9) and (Model IV: HR: 0.7; 95% CI: 0.53–0.94), respectively. (Table 6) (Fig. 1, B) However, the other two identified dietary patterns were not associated with incident HTN. (Table 6) (Fig. 1, C and D).

**Table 6:** Hazard ratio of incident hypertension according to healthy eating index 2015

Dietary pattern	categories	N	N. of cases	Hazard ratio (95% CI)			
				**Model I**	**Model II**	**Model III**	**Model IV**
**Quartiles of HEI 2015**	Q 1	479	84	Ref	Ref	Ref	Ref
	Q 2	337	59	0.84 (0.63, 1.12)	0.94 (0.70, 1.27)	0.98 (0.72, 1.34)	0.97 (0.71, 1.34)
	Q 3	391	82	0.70 (0.53, 0.93)	0.75 (0.57, 0.98)	0.74 (0.55, 0.99)	0.71 (0.52,0.95)
	Q 4	380	69	0.61 (0.46, 0.82)	0.71 (0.52, 0.94)	0.69 (0.51, 0.96)	0.7 (0.51, 0.97)
**Tertiles of plant- based diet**	T1	442	87	Ref	Ref	Ref	Ref
	T2	433	97	0.9 (0.69, 1.16)	1.03 (0.78, 1.34)	1 (0.76, 1.31)	1 (0.76, 1.34)
	T3	420	110	0.69 (0.54, 0.9)	0.71 (0.55, 0.92)	0.68 (0.52, 0.9)	0.7 (0.53, 0.94)
**Tertiles of high protein diet**	T1	400	129	Ref	Ref	Ref	Ref
	T2	426	104	0.82 (0.67, 1.04)	0.84 (0.66, 1.08)	0.89 (0.69, 1.15)	0.94 (0.72, 1.23)
	T3	469	61	0.83 (0.64, 1.09)	0.95 (0.7, 1.29)	1 (0.74, 1.37)	1.1 (0.81, 1.57)
**Tertiles of unhealthy diet**	T1	430	99	Ref	Ref	Ref	Ref
	T2	420	110	1.03 (0.81, 1.32)	1.07 (0.84, 1.37)	1.08 (0.83, 1.39)	1.15 (0.89, 1.5)
	T3	444	85	1.06 (0.82, 1.38)	1.04 (0.8, 1.35)	1.02 (0.78, 1.34)	1.25 (0.9, 1.73)

Model I: Unadjusted; Model II: Adjusted for sex and age; Model III: Adjusted for Model II plus SES, education level, physical activity, T2DM; Model IV: Adjusted for Model III plus BMI and energy intake

Likewise, we observed that among the HEI- 2015 components, the participant who had higher scores of total fruits, fatty acids, and sodium were at lower risk of HTN incident (HR: 0.93; 95% CI: 0.85–0.99), (HR: 0.96; 95% CI: 0.92–0.99), and (HR: 0.96; 95% CI: 0.92–0.99), respectively. Other the HEI- 2015 components had no significant association with HTN incidents. (Table 7).

**Table 7:** Relationship between the HEI- 2015 components and HTN incidence

Components of HEI 2015	Crude	Model 1
	**HR (CI 95%) **^**a**^	**HR (CI 95%) **^**a**^
Total Fruits	0.92 (0.85–0.99)	0.93 (0.85–0.99)
Whole Fruits	0.92 (0.84–1)	0.94 (0.85–1.03)
Total Vegetables	1.02 (0.93–1.12)	1.02 (0.93–1.12)
Greens and Beans	0.98 (0.91–1.07)	0.96 (0.88–1.04)
Whole Grains	1.07 (0.98–1.18)	1.06 (0.96–1.17)
Dairy	0.99 (0.95–1.02)	0.99 (0.95–1.03)
Total Protein Foods	0.95 (0.87–1.04)	0.96 (0.87–1.06)
Seafood and Plant Proteins	1.04 (0.9–1.21)	1.13 (0.97–1.33)
Fatty Acids	0.96 (0.93–0.99)	0.96 (0.92–0.99)
Refined Grains	0.93 (0.84–1.02)	0.91 (0.83–1.01)
Sodium	0.94 (0.91–0.98)	0.96 (0.92–0.99)
Added Sugars	0.98 (0.93–1.03)	1 (0.95–1.05)
Saturated Fats	1.00 (0.97–1.04)	1.01 (0.97–1.05)

^**a**^Adjusted for age, sex, SES, education level, physical activity, T2DM, BMI and energy intake

## Discussion

This large prospective Kurdish population-based study extracted three major dietary patterns using principal component analyses methods, including plant-based, high protein, and unhealthy dietary patterns. We found that greater adherence to HEI 2015 and the plant-based dietary pattern has inversely correlated with the risk of incident HTN. Moreover, among the HEI- 2015 components, a higher score of total fruits, fatty acids, and sodium was associated with a reduced risk of incident HTN. There was no significant association between the other two identified dietary patterns (high protein and unhealthy dietary patterns) and incident HTN.

HTN contributes as one of the most critical risk factors for CVDs and their mortality estimated 874 million adults had an SBP of more than 140 mm Hg or higher in 2015 [21]. Diet modification is an effective strategy to prevent it [22]. Understanding national dietary patterns and their relationship to the risk of chronic non-communicable diseases overcomes the challenge of examining individual foods or nutrients and considering their common effects [23]. In this regard, limited data are available from Kurdish dietary patterns, and to the best of our knowledge, the major dietary patterns were identified and were compared to HEI 2015. The association between them and incident HTN was also evaluated.

This study indicated that higher adherence to HEI 2015 had a preventive effect on incident HTN. Similarly, greater adherence to the plant-based pattern that emphasized the higher intake of vegetables, especially leafy vegetables, fresh fruits, legumes, and whole grains, decreased incident HTN; the high protein diet, however, had no association with incident HTN. According to other studies, high protein diets are associated with higher blood pressure. Therefore, people with high protein diets may have higher blood pressure [1, 6, 24]. A systematic review and meta-analysis of 15 prospective cohort studies showed that higher diet quality characterized by HEI 2005, 2010, and the alternate healthy eating index (AHEI) was significantly related to decreasing all causes of mortality, including CVDs, cancer, and type 2 diabetes [25]. In three populated prospective cohort investigations, greater adherence to various wholesome eating patterns was consistently associated with lower cardiovascular disease risk [26]. A cross-sectional analysis of the Fasa PERSIAN study suggested that the HEI-2015 diet may prevent hypertension [2]. On the contrary, in our study Tehran Lipid and Glucose Study Increasing DASH score, the healthy and unhealthy dietary patterns were not associated with the risk of hypertension [5]. Hu et al. presented that greater following HEI 2015 was significantly lessened CVDs incidence (HR: 0.84; 95% CI: 0.76–0.93) and CVDs mortality (HR: 0.68; 95% CI: 0.58–0.8) [27]. However, HTN was not assessed in these studies. Another systematic review and meta-analysis of 15 randomized clinical trials demonstrated that the vegetarian diet lowered both systolic and diastolic blood pressure compared to the omnivorous diet [28]. A case-cohort study after 1.61 years follow up of 686 vegetarians showed that they were 34% less likely to develop HTN than people who did not follow a vegetarian diet (Odds Ratio(OR): 0.66; 95% CI: 0.5–0.87) [29]. A study by Song et al. showed that women who consumed higher legumes and whole-grain were lower at risk of HTN incidence (HR: 0.77; 95% CI: 0.59–1) [30]. In contrast, the results of Thai cohort study displayed that dietary pattern characterized by greater adherence to vegetables, fruits, soybean products, and milk was not associated with HTN (OR: 0.99; 95% CI: 0.86–1.15) [22]. Likewise, in the Mexican Teachers’ cohort study, there was no significant association between dietary patterns tended to vegetable, fruits, and legumes and HTN (OR: 0.94; 95% CI: 0.84,-1.05) [23].

Plant-based diets are generally higher in terms of diet quality than non-plant-based diets due to their high content of fiber, antioxidants, potassium, and low saturated fat and sodium [31–33]. A plant-based diet prevents incident HTN with beneficial effects on blood viscosity, vasodilation and reduced insulin resistance [28, 34]. Also, with its antioxidant and anti-inflammatory properties and the content of valuable fibres, it changes the colony and strains of the intestinal microflora and improves blood pressure by influencing the renin-angiotensin system and baroreceptors [33, 35]. According to the Lea Borgi study, consuming whole fruits for a more extended period and increasing consumption may reduce the risk of hypertension [36]. In A study conducted in Bangladesh, additionally, found that a higher intake of fruits and vegetables was associated with lower annual pulse pressure and systolic blood pressure, while a higher intake of meat was correlated with higher annual pulse pressure [37]. Based on a systematic review and network meta-analysis, the DASH diet may be the most effective dietary measure for lowering blood pressure between hypertensive and pre-hypertensive patients based on high-quality studies [1]. Hence, researchers have referred to it as the dietary approach to stop hypertension (DASH). To prevent hypertension, it emphasizes limiting sodium intake and increasing potassium intake through vegetables and fruits consumption [38, 39]. A systematic review and meta-analysis on 30 randomized clinical trials observed that adherence to the DASH diet can significantly lower blood pressure in hypertensive and healthy adults [40]. Francisco et al., in their study, reported that adherence to the DASH diet was associated with a significant reduction in blood pressure, even with a short follow-up period [41]. Legumes, fruits and vegetables are enriched antioxidants and essential vitamins, including fibre, vitamin C, potassium, folic acid, magnesium, flavonoids, and carotenoids, and have synergistic effects on lowering blood pressure by improving endothelial function and their antioxidative properties causing vasodilation [42, 43].

Our findings from the HEI 2015 showed that greater score of total fruits, fatty acids, and sodium was associated with a lower risk of incident HTN. In other words, limiting the intake of sodium and adequately unsaturated fatty acids compared to saturated fatty acids and sufficient fruits consumption, reduces the development of HTN. Moreover, in this study, higher adherence to an unhealthy diet was inversely related to HEI 2015 and raised the risk of incident HTN, although this association was insignificant even with controlling potential confounder variables. The study by Mendonça et al. on Spanish adults reported that higher adherence to diet with high content of saturated fats and salt and low content of fresh vegetables and fruits as ultra-processed foods was significantly increased the risk of incident HTN (HR: 1.21; 95% CI: 1.06- 1.37) [44]. The modern diet is associated with processed foods, also known as the Western diet. This pattern is associated with increased intake of calories, sodium, saturated fats, and decreased potassium [45, 46]. High sodium intake is the leading cause of increased hypertension, which inhibits the sodium pump and stimulates the sodium-calcium exchanger type 1 (NCX1), resulting in increased intracellular calcium concentration, which causes vasoconstriction [47, 48]. Additionally, high sodium intake reduces the synthesis of nitric oxide (NO) and increases the plasma level of the endogenous NO inhibitor, which reduces vasodilation [49, 50]. On the other hand, reducing potassium intake and affecting the sodium pump inhibit potassium channels in the cell membrane and increase intracellular calcium, which eventually leads to HTN[51, 52]. All of these factors contribute to the development of HTN [48].

The other two features of HEI 2015 had preventive effects of incident HTN were total fruits and fatty acids. Adequate intake of fruits along with high content of antioxidants, vitamins, minerals and fiber as nutritious diet items play a role in weight control and anthropometric indicators and reduce systemic blood pressure [42, 53]. Unsaturated fatty acids, especially omega-3 fatty acids, play an essential role in vascular vasodilation by increasing the bioavailability of NO and having anti-inflammatory effects [54].

### Strengths and limitations

This study was the first conducted on a large Kurdish population-based study. This present study presented the major dietary pattern using the principal component analyses approach and compared it with HEI 2015. Using validated FFQ that trained nutritionists completed led to a likely reduction of dietary intake reminders compared to self-report dietary intake. We also considered potential confounding variables in all analyses, and we did not include some conditions because of possible changes in their diet. This present investigation, nevertheless, contained some limitations. Since the follow-up period was short, the incidence was low and we could not accomplish analyzes by gender. Likewise, the limited knowledge of food frequency questionnaires to accurately quantify food intake should be considered. Despite the inaccuracy in estimating food intake, a relative comparison between the three groups with distinct levels of adherence may reduce this error. As the final point, although we reckoned many potential confounders, we can not rule out the possibility of residual confounders, including alcohol consumption, which may not be reported due to religious issues.

## Conclusions

To conclude, our findings supported the association between the HEI 2015 and incident HTN. Among the major identified dietary patterns, plant-based and high protein diets were positively correlated with HEI 2015, while the unhealthy diet was inversely correlated with HEI 2015. Similarly, the plant-based diet, and greater score of total fruits, fatty acids, and sodium were preventively associated with incident HTN. Furthermore, the other two dietary patterns had no significant association with incident HTN. Therefore, according to the HEI 2015 recommendations, our study recommends limiting sodium intake and adequate vegetables and fruits consumption.

## Data Availability

Data will be available upon request from the corresponding author.
